# A Rare Case Report on Bowel Obstruction due to Seed Bezoar at the Rectum

**DOI:** 10.1155/crgm/9699209

**Published:** 2025-08-13

**Authors:** Kritick Bhandari, Maria Qadri, Saili Chothe, Anil Pathak, Saujanya Jung Pandey, Sakar Paudel

**Affiliations:** ^1^Department of Internal Medicine, KIST Medical College and Teaching Hospital, Lalitpur, Nepal; ^2^Department of Internal Medicine, Jinnah Sindh Medical University, Karachi, Pakistan; ^3^Department of Internal Medicine, East European University, Tbilisi, Georgia; ^4^Department of Surgery, KIST Medical College and Teaching Hospital, Lalitpur, Nepal; ^5^Department of Internal Medicine, Patan Academy of Health Sciences, Lalitpur, Nepal

**Keywords:** bezoar, case report, gastrointestinal obstruction, rectal seed bezoar

## Abstract

Bezoars are rare gastrointestinal masses composed of indigestible substances, typically found in the stomach but at times occurring at more distal locations. Rectal seed bezoars are particularly unusual and manifest with nonspecific gastrointestinal complaints, predisposing to making the diagnosis difficult and delaying appropriate therapy. We describe a case of a 68-year-old female presenting with acute constipation, tenderness of the rectum, and per-rectal bleeding. The patient had a history of recent consumption of approximately 100 g of sunflower seeds. Examination and imaging of the abdomen revealed findings of obstruction at the rectum. Following the rectal enema, the patient passed a clumped mass of sunflower seeds per rectum, which is consistent with a rectal seed bezoar. She was treated conservatively with stool softeners, antibiotics, ointments, and sitz baths. This case is striking because it illustrates the clinical significance of rectal seed bezoars as a rare but reversible cause of acute constipation. Strong suspicion, meticulous dietary history, and focused examination are needed for diagnosis. Early diagnosis and conservative management can prevent complications and make the employment of invasive diagnostics unnecessary.

## 1. Introduction

Bezoars are an uncommon yet clinically significant condition characterized by the accumulation of indigestible material within the gastrointestinal tract [1]. A bezoar is an indigestible conglomeration trapped in the gastrointestinal tract and takes the name from the core substance that accumulates [2]. In contrast to an impacted hardened fecal matter consisting of a mixture of partially digested/undigested materials, bezoars are specifically formed from an aggregation of a core substance such as seeds or fibers [2]. Their formation is influenced by various anatomical, physiological, and dietary factors, and they can manifest in diverse forms depending on their composition [3]. Although often asymptomatic, bezoars can lead to a wide spectrum of gastrointestinal symptoms and complications, necessitating careful diagnostic evaluation and individualized management. Understanding the underlying mechanisms, risk factors, and treatment modalities is essential for effective diagnosis and intervention. We report a case of a 68-year-old woman who presented with complaints of constipation and was found to have a sunflower seed bezoar at an unusual location: the rectum.

## 2. Case Presentation

A 68-year-old female presented to the emergency department with chief complaints of not being able to pass stool for 1 day. She also complains of per-rectal bleeding and rectal pain. As per the patient, she had used a sharp foreign body (toothpick) to attempt to clear her stool, which led to per-rectal bleed. She does not give a history of nausea/vomiting, abdominal distention, abdominal pain, fever, or recent use of medications. There is no significant past and personal history related to the presenting complaints. While assessing her dietary history, she reported consuming large amounts of sunflower seeds 1 day back, estimated to be around 100 g. The patient reportedly had a normal bowel movement once every day and denies any history of constipation. Her last bowel movement was 2 hours before the consumption of sunflower seeds, and she reported that the stool was of normal consistency. On initial assessments, her vitals were within normal limits and on examination of the abdomen, it was soft, nontender, and not distended. Bowel sounds were heard, and no clinical signs suggestive of any pathology were visible. Examination of the cardiovascular, respiratory, and nervous systems was found to be normal. On digital rectal examination, we found that the stool was impacted in the rectum and it had a firm gritty feeling and also noticed minimum serous fluid oozing from the anal orifice. There was no active bleeding at the time of examination.

**Figure 1:**
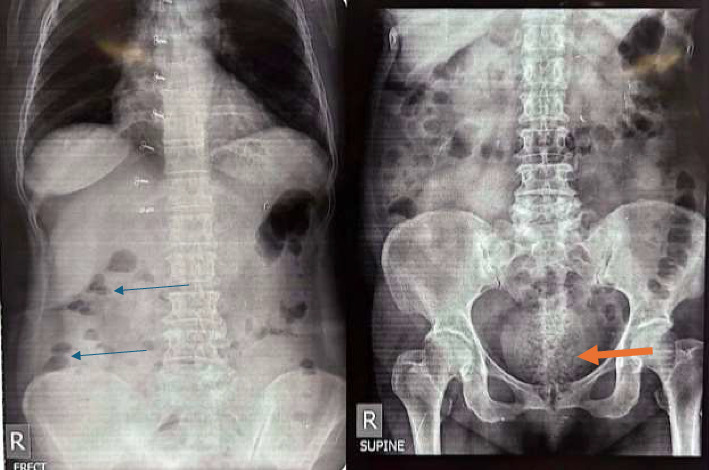
X-ray of abdomen and pelvis showing bowel loaded with stool on the rectal area (orange arrow) and on supine positives, 3-4 air fluid levels were visible suggesting large bowel obstruction.

The routine baseline investigations were sent as part of the initial workup (Supporting File: Supporting Tables 1 and 2). Complete blood count (CBC), renal function test, liver function test, random blood sugar, and urine R/M/E revealed no abnormalities. X-ray of the abdomen and pelvis showed bowel loaded with stool in the rectal area, and 3-4 air-fluid levels were also visible, suggesting large bowel obstruction (Figure 1).

**Figure 2:**
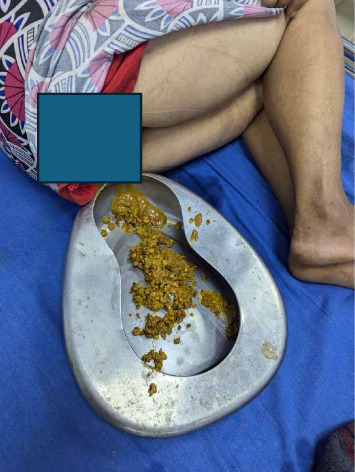
Multiple sunflower seeds are visible on the stool and they have clumped together to form a tight solid mass. No blood staining was present.

The patient was advised to be admitted to the ward for management of her rectal bleeding; however, she declined admission and only requested assistance with the passage of stool. A rectal enema syringe was used, and the patient passed stool within 1 min. On observation, multiple sunflower seeds were visible, and they had clumped together to form a tight, solid mass. No blood staining was present (Figure 2). The patient was diagnosed with acute constipation due to a sunflower seed bezoar. She was also prescribed antibiotic tablet cefixime 200 mg per oral twice daily for 5 days, tablet ornidazole 500 mg per oral for 5 days, syrup lactulose 15 mL per oral at bedtime for 5 days, and ointment Protek (lidocaine, hydrocortisone acetate, zinc oxide, and allantoin) local application twice daily for 7 days. She was also advised to use a sitz bath for 7 days. On follow-up after 7 days, she reported no new complaints and had resumed regular bowel movements.

## 3. Discussion

Bezoars are retained aggregates of indigestible material that accumulate within the gastrointestinal tract. Although they can be found anywhere from the esophagus to the rectum, they most commonly occur in the stomach. Based on their composition, bezoars are classified into four primary types: phytobezoars, composed of undigested fruit and vegetable fibers; trichobezoars, consisting of hair; lactobezoars, formed from undigested milk concretions; and pharmacobezoars, resulting from the accumulation of medications [1, 3]. Seed bezoars represent a distinct subtype of phytobezoars, formed by the accumulation of undigested vegetable or fruit seeds [4, 5].

Bezoars can form in individuals with normal GI anatomy and physiology; those with altered anatomy or motility are at a higher risk [3]. They can occur anywhere in the gastrointestinal tract, from the esophagus to the rectum, and they are most commonly found in the stomach [6]. Because of their small size, seeds tend to pass through the pyloric sphincter and the ileocecal valve and ultimately get stuck in the rectum. Here, they get severely dehydrated, forming a hard and impactful mass. Compared with rectal seed bezoars, gastric bezoars are composed of indigestible fibers like cellulose, which form a glue-like coagulum in the acidic environment of the stomach [7]. Till date, no risk factors have been associated with the development of rectal bezoars [8]. On the contrary, for gastric bezoars, Kement stated that 85.7% of the patients showed one or more predisposing risk factors [7]. Risk factors for gastric bezoar formation include a history of gastric surgery such as partial gastrectomy or vagotomy, neurological conditions like Guillain-Barré syndrome and myotonic dystrophy, and endocrine disorders such as diabetes mellitus (gastroparesis) and hypothyroidism. In addition, excessive persimmon consumption, mastication problems, psychiatric diseases, and pathologies affecting GI motility further increase the risk [3, 5, 8].

In a systematic review by Manatakis et al. [4], they found that 71.9% of total GI bezoar cases were reported from the eastern Mediterranean basin and Middle East, 19% cases were from western Europe and America, and 9.1% cases were reported in Asia [4]. They also found that watermelon seeds were the most consumed cause, accounting for 36% (15/153) of the cases. This was followed by sunflower seeds (19.6%, 30/153) and prickly pear seeds (18.3%, 28/153). Other seeds reported in literature included wild banana seeds, pumpkin seeds, pomegranate seeds, date seeds, tangerine pits, olive pits, lupin seeds, and popcorn kernels [4]. Areca nut chewing, which is a prevalent practice in many parts of Southeast Asia, has also been reported to be a cause of seed bezoars in a case report by Waqas Ali et al. [9].

Gastric bezoars remain asymptomatic for years or may be present with vague, nonspecific symptoms [3, 5]. According to Manatakis et al. [4], 62.7% of the patients (96/153) presented with constipation and 19% of the patients (29/153) reported atypical abdominal or rectal pain along with blood-tinged stools or tenesmus. Likewise, our patient also exhibited signs and symptoms of constipation. According to Kement et al., abdominal pain is the most common symptom and was presented by 95.2% of the patients. A total of 76.2% of the patients showed other dyspeptic symptoms, 69% of the patients had mild to severe nausea and vomiting, 45.2% of the patients lost their appetite, and 11.9% of the patients suffered from significant weight loss [8]. Other reported complications of seed bezoars are bowel perforations and peritonitis [4].

The diagnosis of seed bezoars is primarily based on a thorough clinical history and confirmed through a digital rectal examination [4, 5]. Plain abdominal radiographs show a solid stool mass [4]. Barium studies show mottled or streaked appearance revealing filling defects [3]. CT scans are the gold standard for diagnosing bezoars, providing detailed information on the type, location, degree of obstruction, and potential complications like bowel wall ischemia or perforation [3, 4]. Endoscopy remains the preferred diagnostic technique for bezoars in the esophagus or stomach [2]. Our patient was diagnosed via DRE due to the close proximity of the impacted stool to the anal orifice, and the diagnosis was confirmed via chest X-ray.

Management of bezoars depends on their type and underlying risk factors and involves surgical extraction, endoscopic fragmentation, enzymatic dissolution, gastric lavage, dietary modifications, and prokinetic agents [3]. Manual evacuation and digital fragmentation under general anesthesia to minimize discomfort is the preferred treatment for rectal seed bezoars [4, 9, 10]. Endoscopy alone is often ineffective in extracting a bezoar, as advancing the endoscope beyond the seed mass risks rectal perforation [8, 11]. Phytobezoars are commonly treated with cellulase, papain, or a combination of enzymes like cellulase, cysteine, and metoclopramide [10, 12]. Ladas et al. in 2002 successfully reported the use of Coca-Cola lavage for treatment [13]. Surgical intervention becomes necessary for patients with ileus or refractory bezoars. While open surgical retrieval (laparotomy) was traditionally used, recent studies highlight the benefits of minimally invasive laparoscopy in managing gastrointestinal bezoars [5].

## 4. Conclusion

Seed bezoars, although rare, should be considered in the differential diagnosis of acute constipation, especially when a recent history of excessive seed ingestion is present. Rectal seed bezoars represent an unusual and underreported manifestation, often leading to diagnostic confusion due to their nonspecific symptoms. Our case highlights the importance of detailed history-taking, physical examination, and targeted imaging in identifying bezoars, even at atypical sites like the rectum. Early detection and conservative treatment, like manual disimpaction and rectal enema, can result in a quick resolution without invasive procedures. Clinicians should be aware of regional trends in seed consumption and dietary practices that may make bezoar formation a more relevant diagnosis. Patients can benefit from being informed about the possible gastrointestinal hazards of consuming large amounts of indigestible seeds.

## Data Availability

Data sharing is not applicable to this article as no datasets were generated or analyzed during the current study.

## Nomenclature

GI: Gastrointestinal
CBC: Complete blood count
R/m/e: Routine/microscopy/examination
DRE: Digital rectal examination
CT: Computed tomography

## Supporting Information

Supporting File.docx.
