# Supplementary material for: A Rare Case Report on Bowel Obstruction due to Seed Bezoar at the Rectum
Source: Case Rep Gastrointest Med. 2025 Aug 13;2025:9699209. doi: 10.1155/crgm/9699209 (PMC12367360; doi:10.1155/crgm/9699209)
Supplement: Supporting Information — Additional supporting information can be found online in the Supporting Information section. [file 9699209.f1.docx]

| TEST | RESULT | UNIT | REFERENCE RANGE |
| --- | --- | --- | --- |
| **COMPLETE BOLLOD COUNT** | | | |
| Hemoglobin | 14.2 | gm/dl | 13.5-17.5 |
| Red blood cells | 5.11 | mill/mm3 | 4.5-5.5 |
| White blood cells | 8300 | /ccmm | 4000-11000 |
| Platelets | 280,000 | /ccmm | 150000-450000 |
| Packed cell volume (PCV) | 45.7 | % | 40-50 |
| Mean corpuscular volume (MCV) | 89 | fl | 80-100 |
| Mean corpuscular hemoglobin(MCH) | 29.2 | pg | 27-32 |
| MCHC | 34.5 | g/L | 31.5-35 |
| Red cell Distribution  width (RDW) | 13 | % | 11.6-14.0 |
| Mean platelet volume (MPV) | 10.5 | fl | 6.8-10.9 |
| **DIFFERENTIAL CELL COUNTS** | | | |
| Neutrophils | 68 | % | 45-75 |
| Lymphocytes | 27 | % | 20-45 |
| Monocytes | 7.1 | % | 3-10 |
| Eosinophils | 2.6 | % | 1-8 |
| Basophils | 0.5 | % | 0-1 |
| **ABSOLUTE LEUCOCYTE COUNTS** | | | |
| Absolute Neutrophils | 6.52 | thou/mm3 | 2.00-7.00 |
| Absolute Lymphocytes | 1.68 | thou/mm3 | 1.00-3.00 |
| Absolute Monocytes | 0.56 | thou/mm3 | 0.10-0.60 |
| Absolute Eosinophils | 0.28 | thou/mm3 | 0.02-0.52 |
| Absolute Basophils | 0.03 | thou/mm3 | 0.02-0.10 |

**Supplementary table 1: Complete Blood count of the patient**

| Test | Result | Unit | Reference range |
| --- | --- | --- | --- |
| **RENAL FUNCTION TEST** | | | |
| Urea | 38 | mg/dl | 15-45 |
| Creatinine | 0.89 | mg/dl | 0.3-1.2 |
| Sodium | 139.5 | mmol/L | 135-145 |
| Potassium | 4.1 | mmol/L | 3.5-5.2 |
| **LIVER FUNCTION TEST** |  |  |  |
| Total bilirubin | 0.72 | mg/dl | 0.3-1.2 |
| Direct | 0.31 | mg/dl | 0.1-0.4 |
| Alkaline phosphatse | 49.00 | U/L | 28-115 |
| SGPT(AST) | 15.00 | U/L | 5-40 |
| SGOT(ALT) | 23.00 | U/L | 5-40 |
| Glucose Random | 117 | mg/dl | 70-140 |
| **URINE REPORT** | | | |
| **Urine R/M/E** |  |  |  |
| Colour | Light yellow |  |  |
| Transparency | Clear |  |  |
| Reaction | Acidic |  |  |
| Sugar | Nil |  |  |
| Protein | Nil |  |  |
| **Microscopy** |  |  |  |
| White blood cells | 0-2 | /HPF |  |
| Red blood cells | Nil | /HPF |  |
| Epithelial cells | 0-2 | /HPF |  |
|  |  |  |  |

**Supplementary table 2: Renal Function test, Liver function test and Urine routine examination of the patient**
